# Double Twist and Shout: An Emergency Caused by Torsion of the Ovary and the Wandering Spleen

**DOI:** 10.7759/cureus.71645

**Published:** 2024-10-16

**Authors:** Tim Dreesen, Annelies Kerckhofs, Homa Hosseini, Liesbeth van Bergen, Ali Ramadhan, Stefan Morreel, Maarten Spinhoven, Wiebren A Tjalma

**Affiliations:** 1 Multidisciplinary Breast Clinic – Gynecologic Oncology, Antwerp University Hospital, Edegem, BEL; 2 Faculty of Medicine and Health Sciences, University of Antwerp, Antwerp, BEL; 3 Department of Pathology, Antwerp University Hospital, Edegem, BEL; 4 Department of Obstetrics and Gynecology, Antwerp University Hospital, Edegem, BEL; 5 Department of Abdominal, Pediatric, and Plastic Surgery, Antwerp University Hospital, Edegem, BEL; 6 Department of Abdominal Surgery, Heilig Hartziekenhuis Mol, Mol, BEL; 7 Department of Primary and Interdisciplinary Care, Antwerp Research Group, University of Antwerp, Antwerp, BEL; 8 Department of Radiology, Antwerp University Hospital, Edegem, BEL

**Keywords:** acute abdomen, case report, cellular fibroma, dolichocolon, laparoscopy, ovarian fibroma, splenic infarction, splenic torsion, surgery, wandering spleen

## Abstract

Intermittent severe abdominal pain is a medical emergency with multiple possible underlying causes. This case report describes a 30-year-old female experiencing severe pelvic pain alternating between the left and right lower abdomen. The pain was periodic and very intense. She also experienced intermittent, vague pain in the upper abdomen. Clinical examination and imaging suggested torsion and detorsion of an ovarian fibroma or dysgerminoma. Additionally, a distended dolichocolon was seen in the upper abdomen. Laparoscopy revealed a twisted and enlarged right ovary in the pelvis. In the upper abdomen, a distended dolichocolon was observed along with a twisted, black spleen. The blood vessels of the wandering spleen hung over the colon like a rubber band, causing constriction. The ovary was removed and diagnosed as a cellular fibroma after histopathological examination. Attempts to untwist the spleen laparoscopically were unsuccessful, so a mini-laparotomy was performed to manually untwist it. Afterward, the spleen regained its normal position and color. Due to the significantly elongated dolichosigmoid, which could cause further complications, the affected segment was removed and a reanastomosis was performed. At the end of the procedure, the spleen remained stable in its original position with a healthy coloration. In retrospect, this patient experienced intermittent twisting of the ovary and spleen over an 18-month period, causing severe abdominal pain. The key lessons from this case are to take abdominal pain seriously, thoroughly examine all areas during surgery, and avoid stopping the investigation after identifying a single issue. A critical approach is essential to pinpointing the cause of each specific symptom, as one pathology may not always explain all observed symptoms. This comprehensive approach ultimately saved the patient’s spleen.

## Introduction

Acute abdomen denotes a medical emergency requiring immediate attention, diagnosis, and treatment. Failure to promptly diagnose an acute abdomen can result in fatal consequences. Various causes may precipitate an acute abdomen, including infection, inflammation, vascular occlusion, perforation, obstruction, or torsion. Several of these etiologies necessitate urgent surgical intervention and treatment [1,2]. A wandering spleen is a rare clinical phenomenon, with an overall incidence rate of less than 0.2%. The first report of a wandering spleen was made by Horne in 1667, and the first operative report was made by Martin in 1877 [3]. Ovarian torsion is even less frequent, with an incidence of 9.9 per 100,000 in women of reproductive age (15-45 years) [4]. The first article about ovarian torsion was written by Carl Freiherr von Rokitansky, a pathologist from Vienna, Austria, in 1864 [5]. This case report will discuss an acute abdomen caused by the rare occurrence of a twisted ovarian fibroma accompanied by a simultaneously occurring splenic torsion and their clinical presentations.

## Case presentation

**Figure 1:**
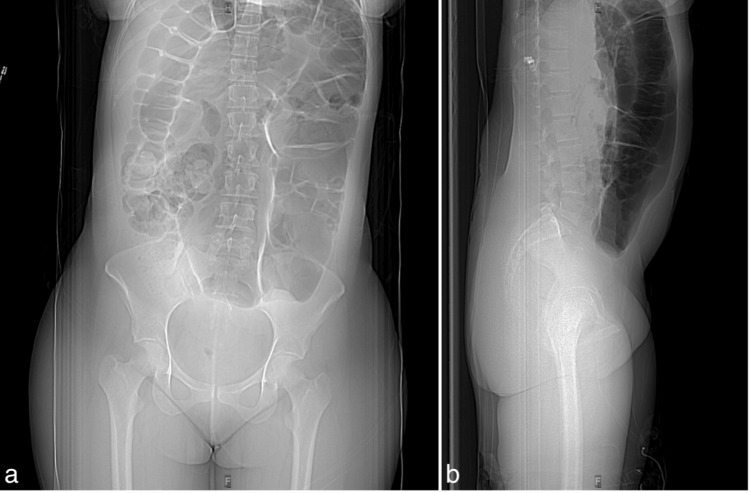
CT abdomen, low dose without IV contrast (Revolution CT, GE Healthcare®) Coronal (a) and sagittal (b) scout images showing an air-filled distended sigmoid colon. CT: computed tomography, IV: intravenous

This case involves a 30-year-old female, G2P2A0, with an unremarkable medical history who presented to the emergency department with alternating periods of severe abdominal pain. The pain initially started as a stabbing sensation in the right flank and had been radiating to the right iliac and suprapubic regions for the past two days. She reported chills without fever, along with an urge to move and pollakisuria. Given her symptoms, a urinary tract infection (UTI), nephrolithiasis, cholecystitis, chole(docho)lithiasis, normal or ectopic pregnancy, appendicitis, inflammatory bowel disease, and possibly irritable bowel syndrome were suspected. A physical examination revealed no abnormalities except for mild abdominal distention. Blood analyses were normal, and human chorionic gonadotropin (hCG) was negative. Urine culture and microscopy were also negative, effectively ruling out a UTI. An abdominal CT scan did not show any signs of nephrolithiasis. However, it did reveal severe aerocoly, indicating the accumulation of gas in the colon, resulting in colon distention. The aerocoly was mainly observed in the descending and sigmoid colon, with significant sigmoid distention up to 80 mm and two areas of constriction (Figure 1). No intestinal obstruction was noted. The patient was treated with analgesics and nitrofurantoin, which alleviated her pain. She was eventually discharged from the hospital after her condition improved.

**Figure 2:**
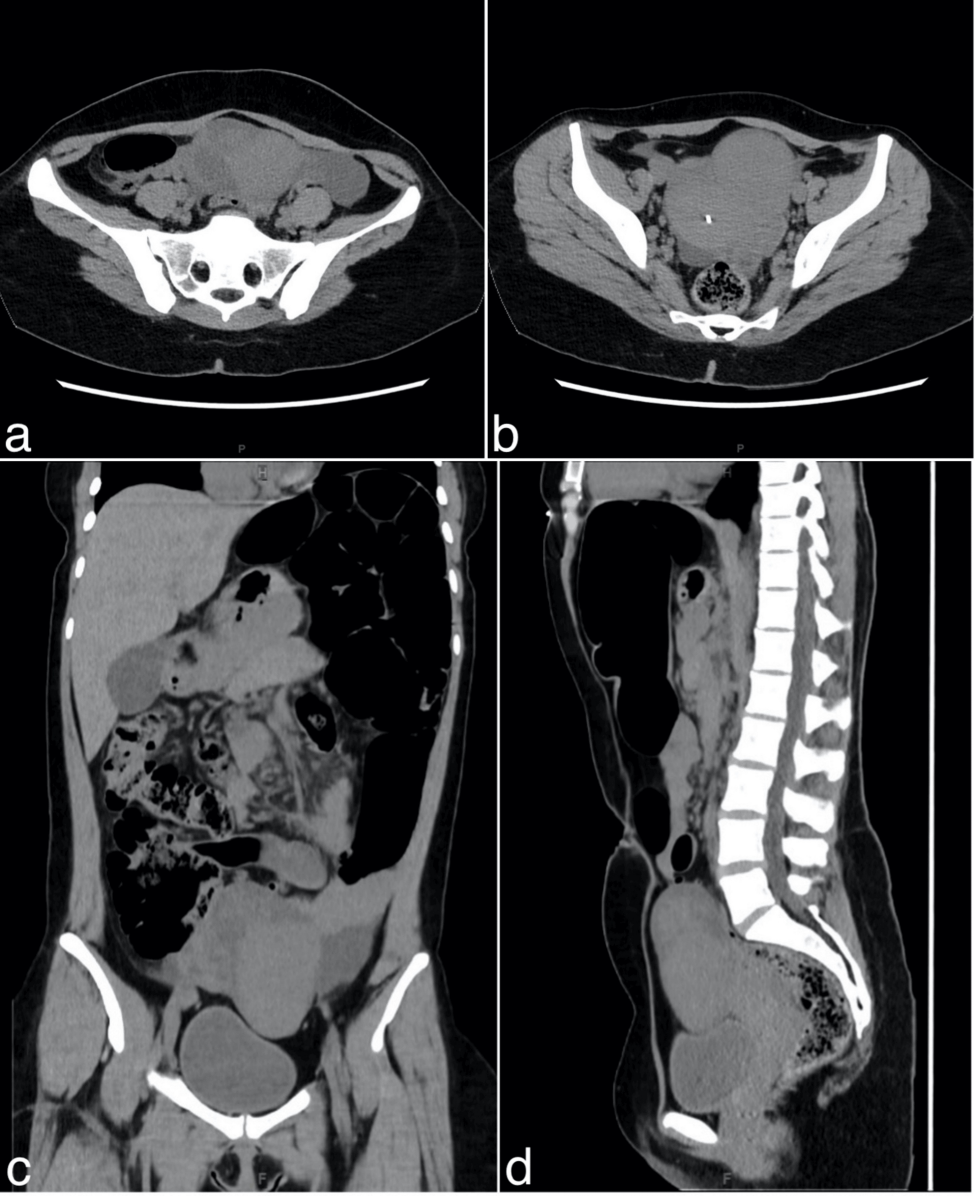
CT abdomen, low dose without IV contrast (Revolution CT, GE Healthcare®) Axial (a, b), coronal (c), and sagittal (d) CT images show a soft tissue mass anterior, cranial, and just to the left of the uterus with surrounding free abdominal fluid. CT: computed tomography, IV: intravenous

Six months later, she returned to the same emergency department with severe abdominal pain. During the intervening months, she reported having recurrent episodes of stabbing pain in the right iliac region, occasionally extending to the left iliac and lumbar regions. Additionally, she experienced diffuse abdominal pain that was difficult to localize. Upon physical examination, her abdomen was soft, non-tender, and slightly distended. The absence of psoas-, obturator-, and rovsing signs made appendicitis less likely [6]. Unlike the first CT scan, a new scan revealed a soft tissue mass located in the pelvic cavity (Figure 2). Interestingly, the mass was positioned slightly toward the left of the pelvic cavity, which curiously was contradictory to her predominant pain in the right iliac region. The CT scan also showed persistent aerocoly and a dolichosigmoid with a caliber jump at the level of the proximal sigmoid, suggesting a structural anomaly but not indicative of an obstruction. Free fluid was also detected in the pelvis, left paracolic gutter, and splenic area, which was not present in earlier scans.

**Figure 3:**
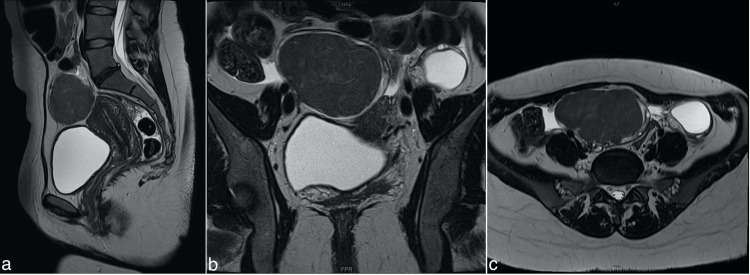
MRI pelvis (1.5 T Magnetom Sola, Siemens Healthineers®) Sagittal (a), coronal (b), and axial (c) TSE T2 images in high resolution show a soft tissue mass anterior, cranial, and just to the right of the uterus with surrounding free abdominal fluid. MRI: magnetic resonance imaging

**Figure 4:**
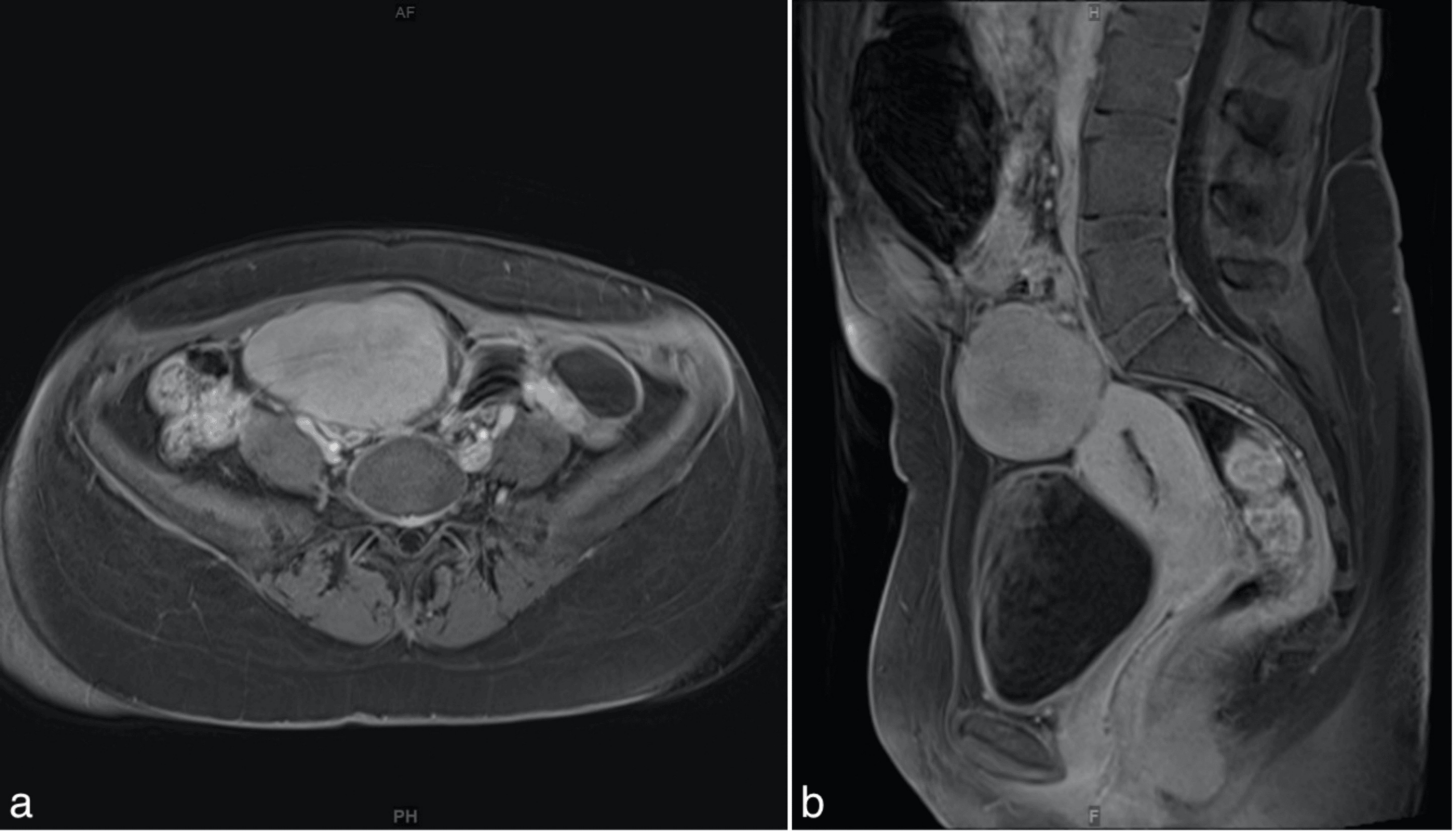
MRI pelvis (1.5 T Magnetom Sola, Siemens Healthineers®) Axial (a) and sagittal (b) fat-suppressed VIBE T1 images in high resolution after IV Gadolinium contrast injection show a homogeneous enhancement of the soft tissue mass. MRI: magnetic resonance imaging

An abdominal MRI scan was performed to further evaluate the soft tissue mass (Figure 3). The MRI scan revealed a mass originating from the right ovary (55 x 60 x 66 mm) and showing homogenous enhancement after gadolinium injection, raising suspicion of a fibroma or dysgerminoma (germ cell tumor) (Figure 4). Interestingly, the MRI scan showed that the mass had shifted from its earlier position on the left to move toward the right side of the pelvic cavity. This shift in the position of the right ovary mass might be due to its mobility within the pelvic cavity, leading to sporadic torsion and detorsion, which could explain the patient’s recurring pain in both the left and right iliac regions. The pain on the left side of her abdomen might be referred to as pain due to this mobility. Furthermore, the left ovary displayed multiple ovarian cysts, with the largest measuring up to 33 x 30 x 34 mm, without suspicious papillary projections.

**Figure 5:**
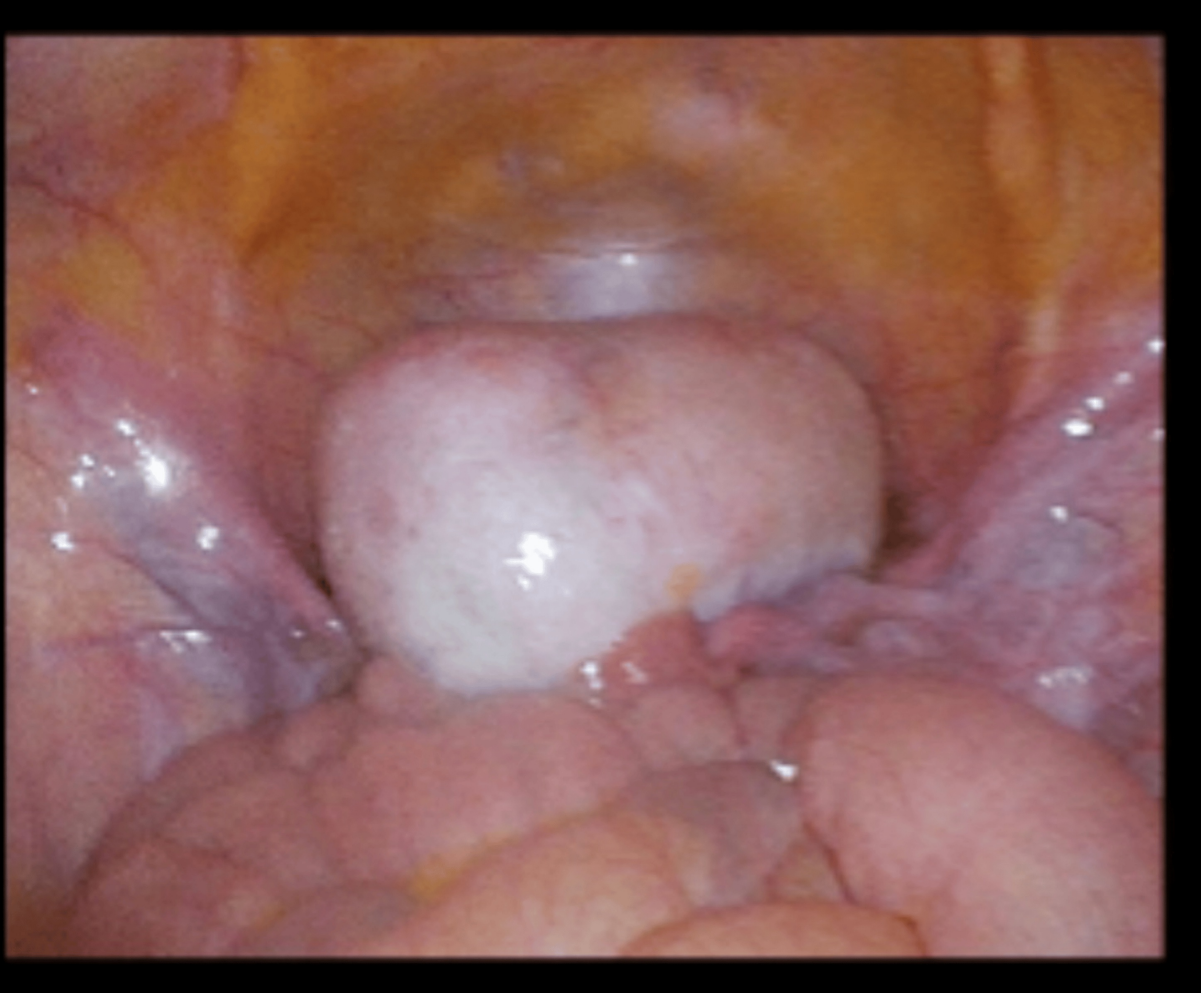
A twisted, enlarged but smooth ovarian mass central in the pelvis (measuring 90 x 50 x 60 mm)

**Figure 6:**
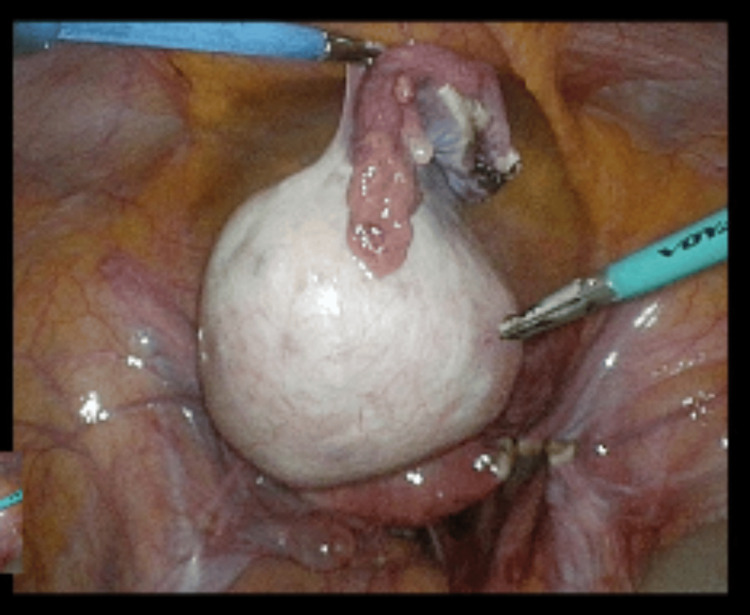
Ovarian mass, ovary, and tube right after resection

**Figure 7:**
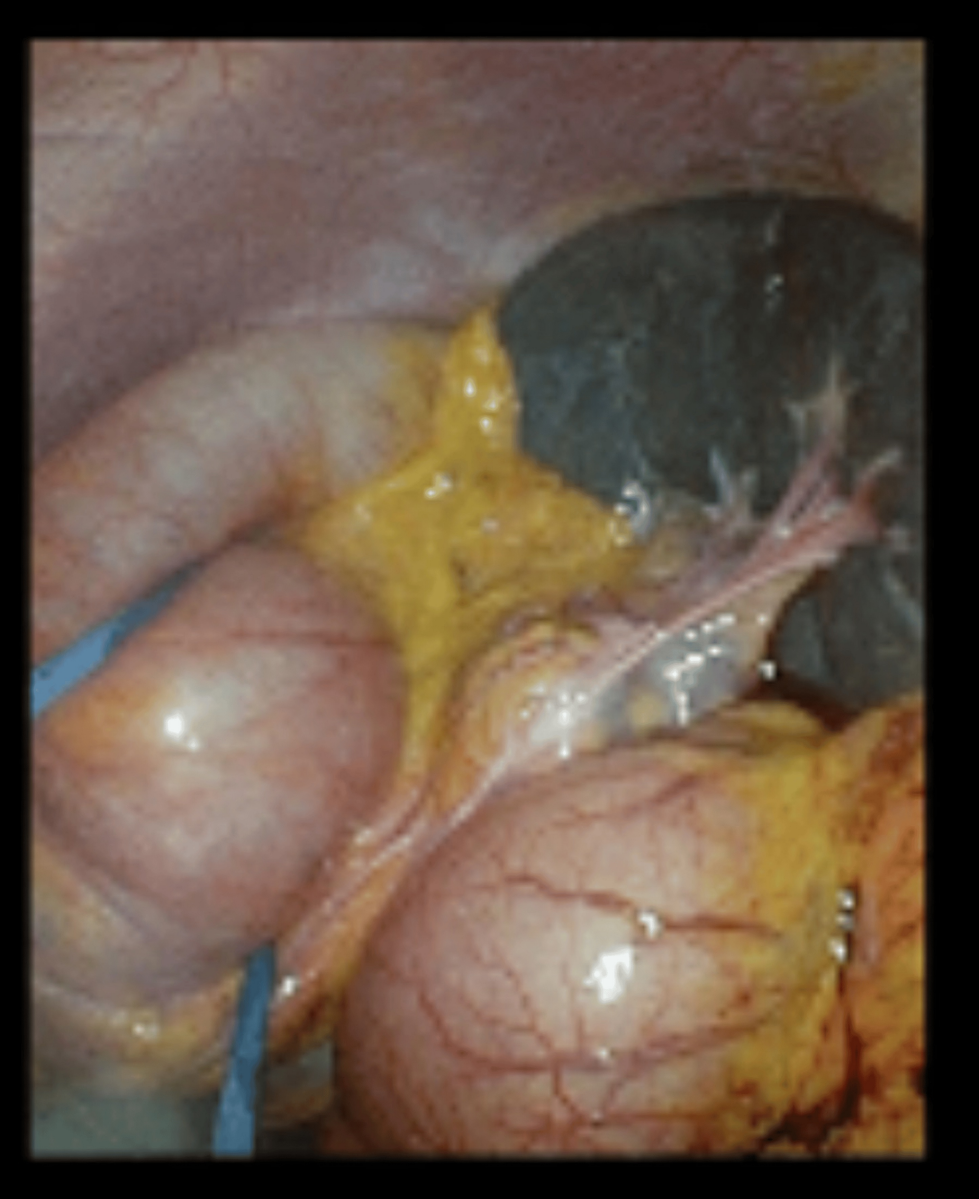
Twisted spleen with its blood vessels hung over the distended dolichocolon like a rubber band, causing a constriction

**Figure 8:**
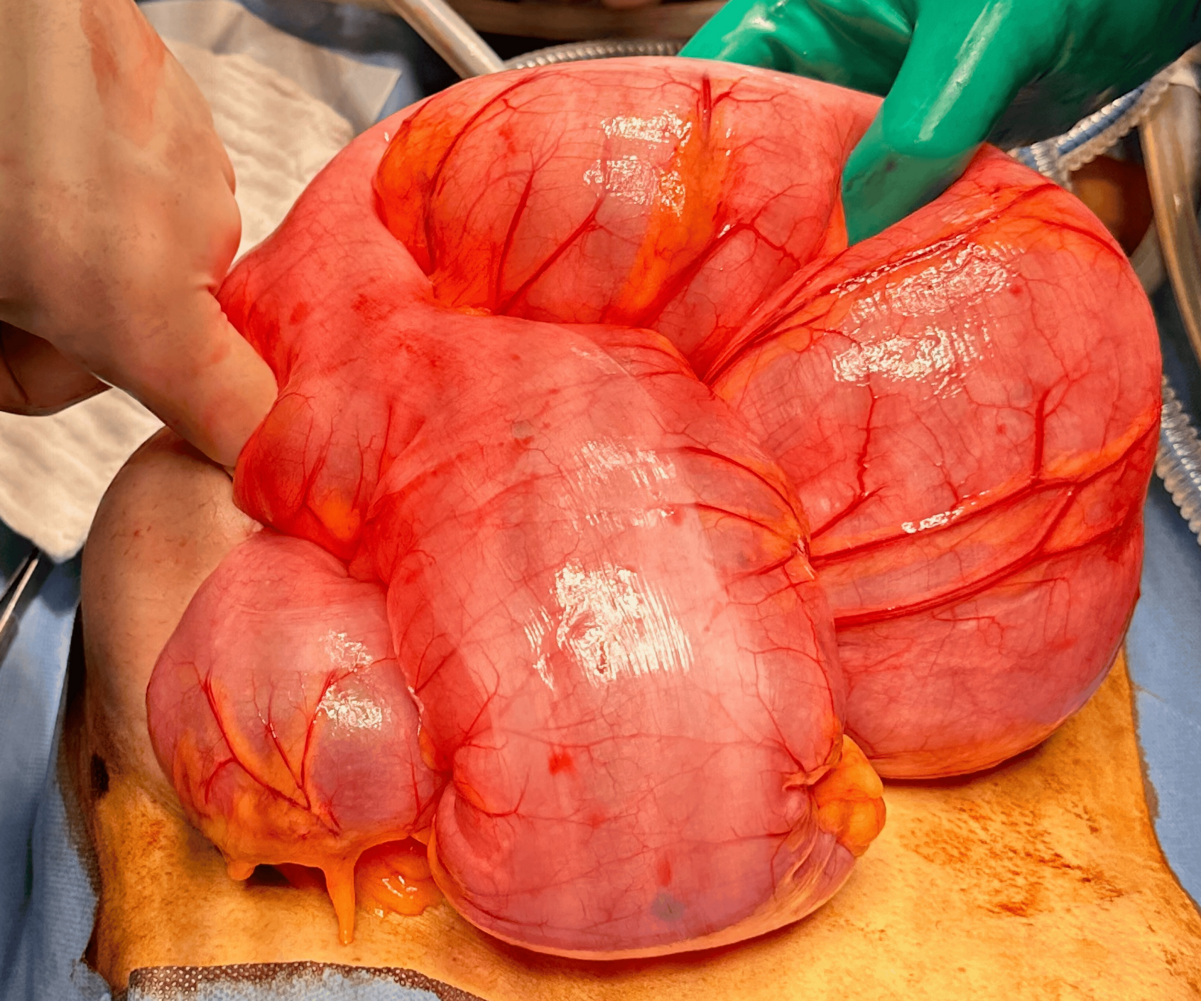
View of the significantly elongated dolichosigmoid after untwisting of the spleen

**Figure 9:**
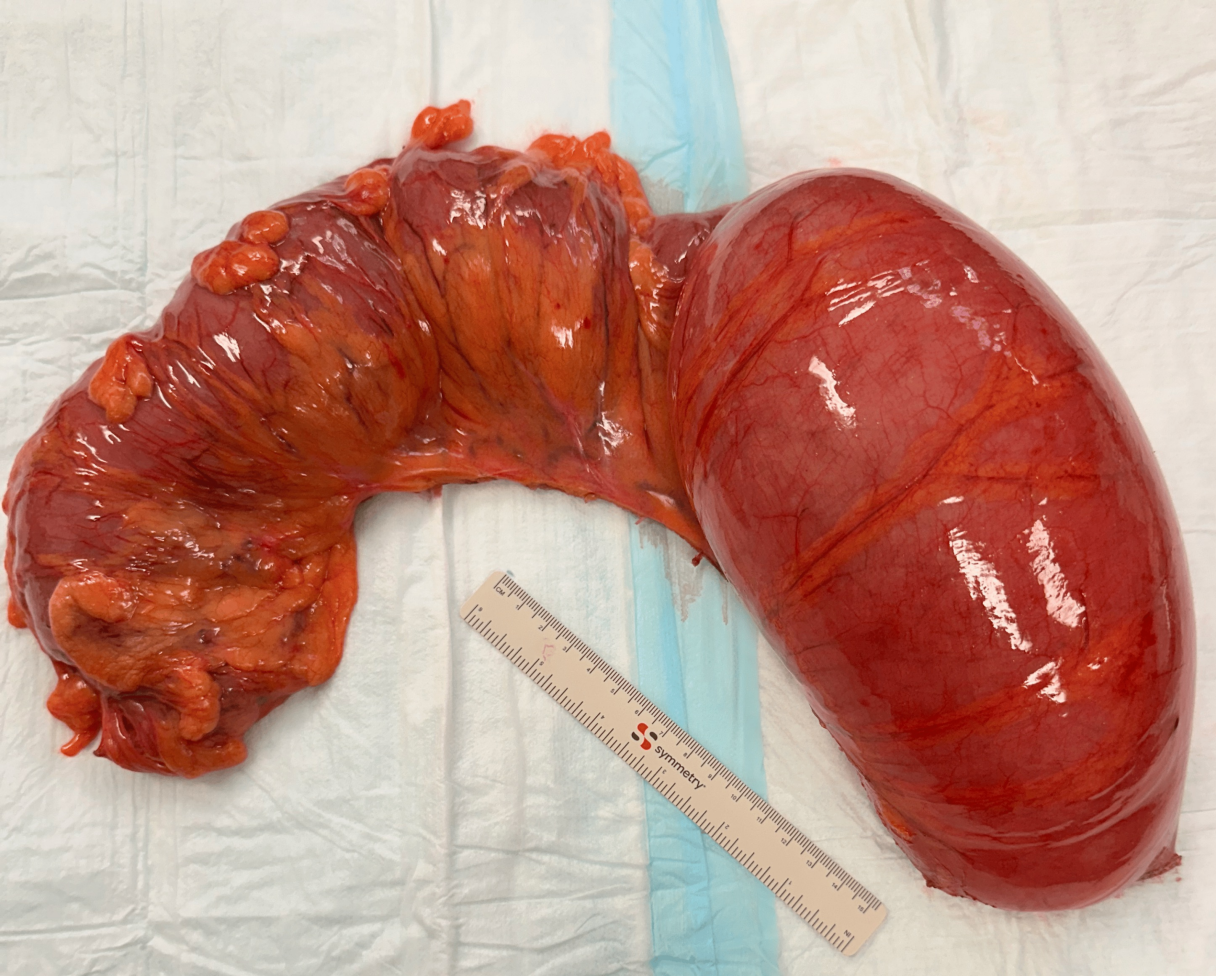
Removed segment of the distended dolichocolon

Blood markers for germ cell tumors (LDH, CA-125, alpha-fetoprotein) were normal. The patient was counseled regarding an exploratory laparoscopy, excision of the mass, and ovarian preservation. Informed consent for the procedure was obtained. Intraoperatively, a twisted, enlarged but smooth ovarian mass, measuring 90 x 50 x 60 mm, was observed in the pelvis (Figure 5). The mass was detached from the infundibulopelvic ligament (suspensory ligament of the ovary) and the proper ovarian ligament (utero-ovarian ligament) and was then removed (Figure 6). Interestingly, the laparoscopy revealed that the spleen, typically located just below the diaphragm on the left side, was unusually positioned, appearing lower and more central in the abdomen. Additionally, the spleen had a black color and a forward tilt. A distended dolichocolon was also noticeable. The blood vessels of the black spleen hung over the colon like a rubber band, causing a constriction (Figure 7). An abdominal surgeon was consulted, and the patient was subsequently diagnosed with a torsion of a wandering spleen. Attempts were made to detort the spleen laparoscopically but were unsuccessful. A mini-laparotomy was performed to manually untwist the spleen, after which it regained its normal position and color. Given the presence of a significantly elongated dolichosigmoid (Figure 8), which could cause further complications, the affected segment was removed (Figure 9), and a reanastomosis was performed with the assistance of the consulted abdominal surgeon. At the end of the procedure, the spleen remained stable in its original position with a healthy coloration. Therefore, no splenopexy or splenectomy was performed. At present, six months after surgery, the patient is doing well and has reported no recurrence of pain.

**Figure 10:**
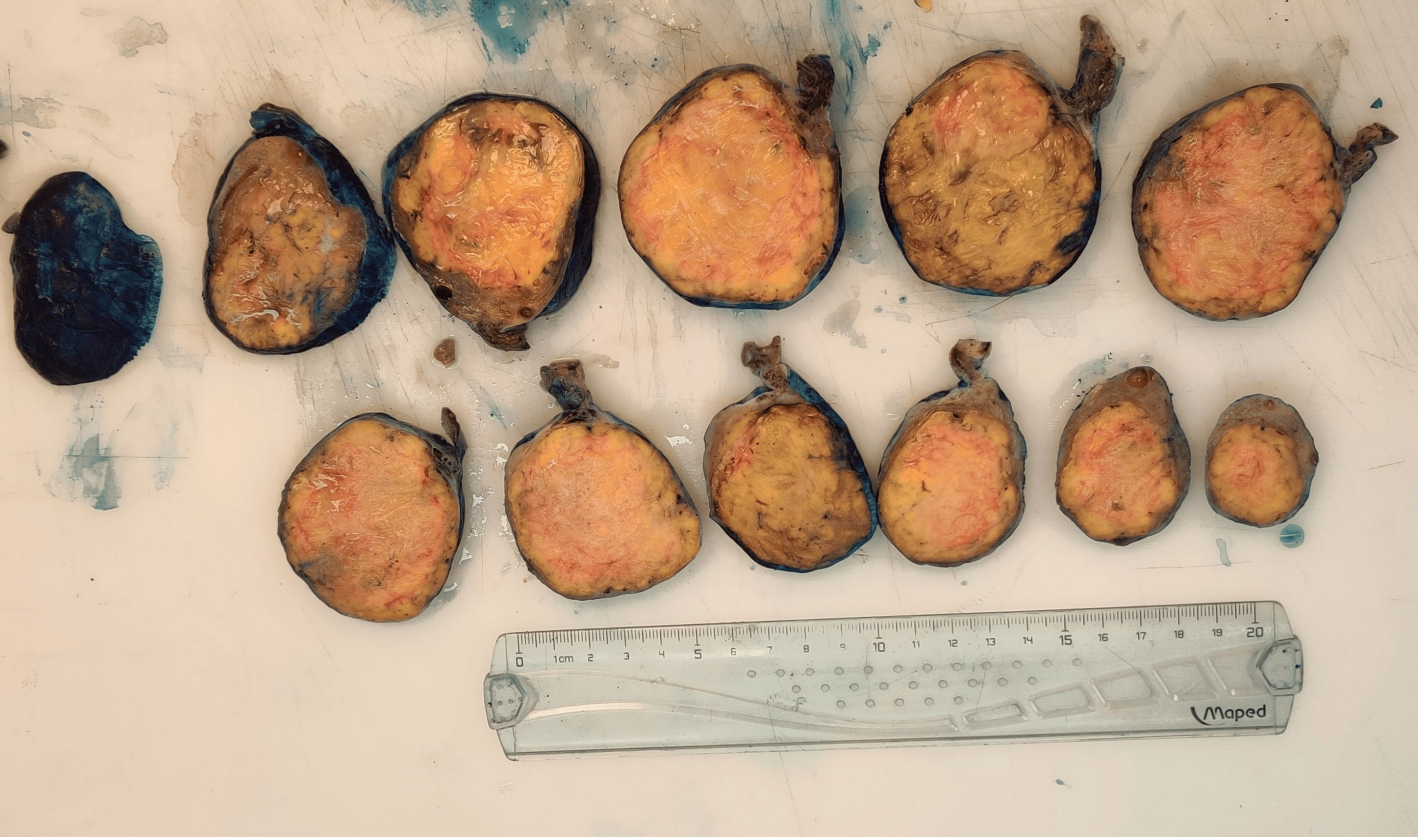
Macroscopical view after formalin fixation A yellow, firm, and encapsulated tumor is seen at the cut surface. It is a rather homogeneous tumor without foci of necrosis or hemorrhage. Notice the intact fibrous capsule around the lesion.

**Figure 11:**
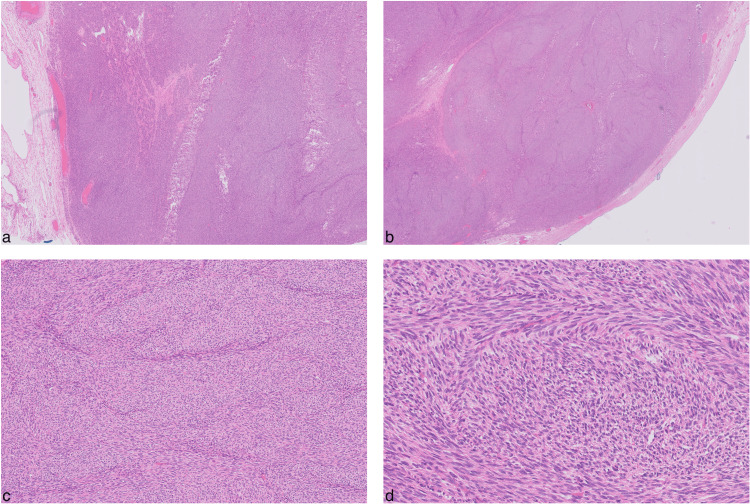
Microscopical histopathological features Microscopical histopathological slides (low (a,b) and high-power view (c,d)) depicting characteristic features of cellular fibroma, including spindle-shaped cells with scant cytoplasm and elongated nuclei arranged in intersecting fascicles, evident collagenous stroma, and minimal mitotic activity. Stained with H&E. H&E: hematoxylin and eosin

**Figure 12:**
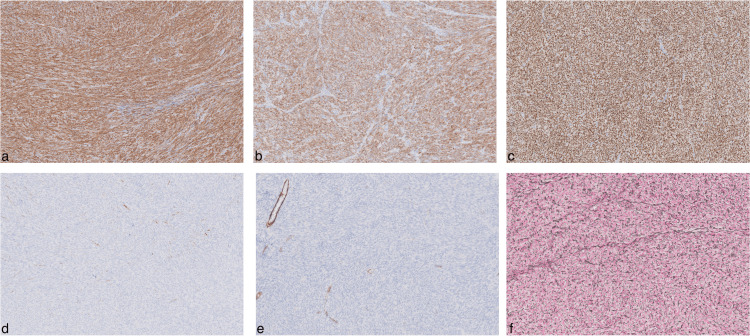
Immunohistochemical staining An immunohistochemical slide demonstrating positivity for SMA (a), inhibin (b), and PR (c) in cellular fibroma tissue. Negativity for desmin (d) and caldesmon (e) further supports the diagnosis. The reticulin stains around every single cell (f). SMA: smooth muscle actin, PR: progesterone receptor

Upon examination, the excised ovarian mass was almost completely taken over by a dense, yellow, and well-circumscribed structure (Figure 10). No extracapsular invasion, necrosis, hemorrhage, or cystic degeneration was observed. Histopathological analysis with hematoxylin and eosin (H&E) staining showed a substantial lesion encased within a fibrous capsule. The cells were predominantly spindle-shaped and arranged in intersecting fascicles, with occasional cells having oval nuclei, evident collagenous stroma, and minimal mitotic activity (two mitotic figures per 2 mm²) (Figure 11). Immunohistochemistry revealed strong positivity for smooth muscle actin (SMA), inhibin, and progesterone receptor (PR), along with negative staining for desmin and caldesmon, and the distinct staining pattern of reticulin further supports the diagnosis of a benign cellular fibroma (Figure 12).

## Discussion

Patients with an acute abdomen typically present with sudden-onset abdominal pain, often accompanied by nausea and vomiting [1]. The diagnostic approach to an acute abdomen relies primarily on the clinical assessment, consisting of the patient’s medical history and physical examination. It is this clinical assessment that determines further diagnostic investigations, enabling an adequate and timely treatment. These additional diagnostics may include laboratory tests, imaging studies (ultrasonography, X-ray, CT, or MRI scans), and interventional procedures such as laparoscopy [7-9].

An adnexal mass and an acute abdomen have several differential diagnoses, which vary according to age. The clinical presentation, age, and biomarkers provide guidance to the most likely options (Table 1). An acute abdomen itself presents a wide array of potential differential diagnoses, particularly in females (Table 2). While most masses or tumors in children and adolescents are benign, their presentations are more challenging and require specialized expertise [10].

**Table 1:** Abdominal masses divided according to age groups with tumor markers, clinical presentation, clinical examination, laboratory testing, and imaging AFP (alpha-fetoprotein): elevated in yolk sac tumors, β-hCG (beta-human chorionic gonadotropin): elevated in choriocarcinoma and mixed germ cell tumors, LDH (lactate dehydrogenase): elevated in dysgerminomas, CA-125 (cancer antigen 125): elevated in epithelial ovarian cancer, HE4 (human epididymis protein 4): elevated in epithelial ovarian cancer, CEA (carcinoembryonic antigen): elevated in gastrointestinal cancers and sometimes in ovarian cancer, CA 19-9: elevated in pancreatic and gastrointestinal cancers, CRP (C-reactive protein): elevated in inflammatory conditions, inhibin: elevated in granulosa cell tumors and mucinous ovarian tumors, PID: pelvic inflammatory disease, CBC: complete blood count, CT: computed tomography, MRI: magnetic resonance imaging, PET: positron emission tomography

Age Group	Masses	Tumor Markers	Clinical Presentation	Clinical Examination	Laboratory Testing	Imaging
Infancy and prepubertal (0-13 years)	Functional cyst	-	Asymptomatic, abdominal distension	Palpable mass	CBC, urinalysis	Ultrasound
	Germ cell tumor	AFP, β-hCG	Abdominal mass, pain, distension, precocious puberty	Palpable mass, tenderness	AFP, β-hCG	Ultrasound, CT/MRI
Adolescent (10-19 years)	Functional cyst	-	Asymptomatic, abdominal pain, menstrual irregularities	Pelvic mass, tenderness	CBC, urinalysis	Ultrasound
	Germ cell tumor	AFP, β-hCG	Abdominal mass, pain, menstrual irregularities	Pelvic mass, tenderness	AFP, β-hCG, LDH	Ultrasound, CT/MRI
	Benign cystic teratoma/other germ cell tumors	AFP, β-hCG, LDH	Abdominal mass, pain	Pelvic mass, tenderness	AFP, β-hCG, LDH	Ultrasound, CT/MRI
	Obstructing vaginal or uterine anomalies	-	Abdominal pain, urinary symptoms	Pelvic mass, structural anomalies	CBC, urinalysis	Ultrasound, MRI
	Epithelial ovarian tumor	CA-125, CEA	Abdominal mass, pain, bloating	Pelvic mass, tenderness	CA-125, CEA	Ultrasound, CT/MRI
Reproductive (15-49 years)	Functional cyst	-	Asymptomatic, pelvic pain, menstrual irregularities	Pelvic mass, tenderness	CBC, urinalysis	Ultrasound
	Pregnancy, ectopic pregnancy	-	Abdominal pain, amenorrhea, vaginal bleeding	Abdominal tenderness, adnexal mass	β-hCG	Ultrasound
	Uterine fibroids	-	Heavy menstrual bleeding, pelvic pain, pressure symptoms	Enlarged uterus, palpable mass	CBC, urinalysis	Ultrasound, MRI
	Epithelial ovarian tumor	CA-125, HE4, inhibin	Abdominal mass, pain, bloating, weight loss	Pelvic mass, tenderness	CA-125, HE4, inhibin	Ultrasound, CT/MRI
	Mature cystic teratoma	AFP, β-hCG, LDH	Abdominal mass, pain	Pelvic mass, tenderness	AFP, β-hCG, LDH	Ultrasound, CT/MRI
	Tubo-ovarian masses (acute/chronic)	CA-125, CRP (for inflammation)	Acute pain, fever, pelvic mass	Pelvic mass, tenderness, fever	CA-125, CRP	Ultrasound, CT
	PID	-	Lower abdominal pain, fever, vaginal discharge	Cervical motion tenderness, adnexal tenderness	CBC, CRP, ESR, STD tests	Ultrasound, MRI
Peri-menopausal (45-55 years)	Fibroids	-	Heavy menstrual bleeding, pelvic pain, pressure symptoms	Enlarged uterus, palpable mass	CBC, urinalysis	Ultrasound, MRI
	Epithelial ovarian tumor	CA-125, HE4, inhibin	Abdominal mass, pain, bloating, weight loss	Pelvic mass, tenderness	CA-125, HE4, inhibin	Ultrasound, CT/MRI
	Functional cyst	-	Asymptomatic, pelvic pain	Pelvic mass, tenderness	CBC, urinalysis	Ultrasound
Post-menopausal (>51 years)	Ovarian tumor (malignant or benign)	CA-125, HE4, CEA, inhibin	Abdominal mass, pain, bloating, weight loss	Pelvic mass, tenderness	CA-125, HE4, CEA, inhibin	Ultrasound, CT/MRI
	Bowel, malignant, or inflammatory	CEA, CA 19-9	Abdominal pain, changes in bowel habits, weight loss	Abdominal tenderness, palpable mass	CEA, CA 19-9	CT, colonoscopy
	Metastases	Dependent on primary tumor markers	Abdominal mass, pain, systemic symptoms	Variable, depending on primary tumor	Dependent on primary tumor markers	CT, PET scan

**Table 2:** Major differential diagnoses of an acute abdomen in a female with clinical features and diagnostic considerations PID: pelvic inflammatory disease, AAA: abdominal aortic aneurysm, hCG: human chorionic gonadotropin, CT: computed tomography, MRI: magnetic resonance imaging, HIDA: hepatobiliary iminodiacetic acid, PET: positron emission tomography

Categories	Conditions	Clinical Features	Diagnostic Considerations
Gastroenterology	Appendicitis	Right lower quadrant pain, nausea, vomiting, subfebrile/fever	Elevated white blood cell count, abdominal ultrasound, or CT scan
	Diverticulitis	Left lower quadrant pain, fever, nausea, change in bowel habits	Abdominal CT scan, physical examination
	Abscess diverticular	Left lower quadrant pain, fever, nausea	Abdominal CT scan, physical examination
	Gastroenteritis	Diffuse abdominal pain, diarrhea, vomiting, fever	Clinical history, stool studies, and supportive care
	Gastric ulcer perforation	Severe abdominal pain, peritonitis symptoms, nausea	Abdominal X-ray, CT scan, physical examination
	Bowel obstruction	Crampy abdominal pain, distension, vomiting, constipation	Abdominal X-ray, CT scan
	Intussusception	Severe abdominal pain, red currant jelly stools, vomiting	Abdominal ultrasound, CT scan
	Obstructed ventral hernia	Sudden abdominal pain, nausea, vomiting, visible bulge (if lean)	Physical examination, abdominal ultrasound, CT scan
	Cholecystitis	Right upper quadrant pain, fever, nausea, vomiting	Abdominal ultrasound, possibly HIDA scan
	Pelvic/wandering spleen	Rare condition, possibly splenomegaly in the pelvis	Abdominal ultrasound, CT scan, physical examination
Gynecology	Ectopic pregnancy	Lower abdominal pain, amenorrhea, vaginal bleeding	Positive pregnancy test, transvaginal ultrasound
	Molar pregnancy	Abnormal vaginal bleeding, uterine enlargement, high hCG levels	Transvaginal ultrasound, serum hCG levels
	Endometriosis	Chronic pelvic pain, dysmenorrhea, dyspareunia	Pelvic examination, transvaginal ultrasound, laparoscopy
	PID	Lower abdominal pain, fever, vaginal discharge, dysuria	Pelvic examination, transvaginal ultrasound, laboratory tests
	Ovarian cyst rupture	Sudden onset of severe abdominal pain, nausea, vomiting	Transvaginal ultrasound, pelvic examination
	Ovarian torsion	Sudden onset of severe unilateral pain, nausea, vomiting	Transvaginal ultrasound with Doppler, physical examination
	Uterine fibroids	Pelvic pain, heavy menstrual bleeding, pressure symptoms	Pelvic ultrasound, MRI
	Hematometra	Abdominal or pelvic pain, difficulty with menstrual flow	Pelvic ultrasound, physical examination
	Pyometra	Severe pelvic pain, fever, purulent vaginal discharge	Pelvic examination, ultrasound, or CT scan
Nephrology / Urology	Kidney stones	Severe flank pain radiating to the groin, hematuria	Abdominal or renal ultrasound, CT scan
	Urinary retention	Abdominal distension, difficulty urinating	Bladder ultrasound, physical examination
	Pelvic kidney	Abdominal pain, possible palpable mass, urinary symptoms	Renal ultrasound, CT scan
Oncology	Colorectal cancer	Change in bowel habits, rectal bleeding, weight loss	Colonoscopy, biopsy, CT scan
	Bowel cancer	Similar to colorectal cancer	Colonoscopy, biopsy, CT scan
	Mesenteric cancer	Abdominal pain, weight loss, possibly palpable mass	Abdominal ultrasound, CT scan, biopsy
	Ovarian cancer	Abdominal bloating, pelvic pain, abnormal vaginal bleeding	Transvaginal ultrasound, CT scan, CA-125 levels
	Endometrial cancer	Abnormal uterine bleeding, pelvic pain, postmenopausal bleeding	Endometrial biopsy, transvaginal ultrasound, CT scan
	Kidney cancer	Flank pain, hematuria, palpable mass	Renal ultrasound, CT scan, MRI
	Bladder cancer	Hematuria, dysuria, pelvic pain	Urinalysis, cystoscopy, imaging studies
	Neuroblastoma	Abdominal mass, weight loss, possibly paraneoplastic syndromes	Abdominal ultrasound, CT scan, biopsy
	Hodgkin lymphoma	Abdominal pain, fever, night sweats, weight loss	Lymph node biopsy, CT scan, PET scan
Cardiovascular	AAA	Severe abdominal or back pain, pulsatile mass	Abdominal ultrasound, CT scan

In this case, the suspicion of ovarian torsion was at the forefront. After pathological examination, the enlarged ovary was diagnosed as an ovarian fibroma. The term “ovarian fibroma” was originally coined by Astrue in 1743 [11]. Ovarian fibromas, commonly referred to as cellular fibromas of the ovary, are benign solid tumors that originate from the stromal component of the ovary. They represent the most prevalent benign solid ovarian tumor, accounting for 1-4% of all ovarian tumors [12,13]. Along with Brenner tumors, ovarian thecomas, and fibrothecomas, ovarian fibromas are classified as sex-cord stromal cell tumors of the ovary [14]. Ovarian fibromas are typically unilateral and can range in size from microscopically small to extremely large [13]. While they are most commonly observed in peri- and postmenopausal patients, they can also occur in younger women, although rarely in children. Table 3 provides a comprehensive list of gynecologic differential diagnoses that should be considered in a 30-year-old female presenting with an ovarian mass [15,16].

**Table 3:** Gynecologic differential diagnoses of an ovarian mass

Categories	Conditions	Examples/Subtypes
Benign	Functional cyst	-
	Endometrioma	-
	Paratubal cysts	-
	Hydrosalpinx	-
	Ectopic pregnancy	-
	Ovarian torsion	-
	Leiomyoma	-
	Germ cell tumor	Mature teratoma
	Sex-cord stromal tumor	Fibroma, thecoma, fibrothecoma, Brenner tumor
	Epithelial tumor	Serous cystadenoma, mucinous cystadenoma
Malignant	Germ cell tumor	Dysgerminoma
	Sex-cord stromal tumor	Granulosa cell tumor
	Epithelial tumor	Epithelial carcinoma
	Metastatic tumors	-

Ovarian fibromas are usually asymptomatic and are often discovered incidentally. When symptomatic, they typically present with abdominal pain in the iliac regions [17]. In about 1% of cases, ovarian fibromas are associated with ascites, pleural effusion, and elevated serum levels of CA-125. This is called Meigs syndrome, which may lead to misdiagnosis of a malignant ovarian tumor. Likewise, Pseudo-Meigs syndrome, or Tjalma syndrome, which includes ascites, pleural effusion, and elevated serum CA-125 in patients with systemic lupus erythematosus, is usually associated with a malignancy and can present as an acute abdomen [18]. The recommended treatment for an ovarian mass is surgical removal, preferably through either laparoscopy or laparotomy, with a preference for preserving fertility [13]. After removal, a thorough analysis using immunohistochemical markers such as desmin, inhibin, and SMA is crucial for accurately classifying and differentiating ovarian fibromas from other ovarian neoplasms [19].

Wandering spleen, also known as hypermobile spleen, is a condition characterized by the spleen becoming hypermobile due to the elongation or maldevelopment of the spleen’s suspensory ligaments. Due to the hypermobility of the spleen, the spleen can become twisted, eventually resulting in splenic torsion. Splenic torsion is a rare yet critical differential diagnosis in patients presenting with an acute abdomen, as it can rapidly progress to splenic infarction [20].

The cause of the splenic torsion in our patient is multifactorial. On one hand, the patient had a wandering spleen, and on the other hand, she had a dolichocolon. Due to this wandering spleen, the spleen relocated itself from its usual position, next to the left colic flexure, to in front of the left colic flexure, where it eventually became knotted to the sigmoid colon. Normally, this phenomenon would not cause immediate major complications for the spleen, except for an increased risk of splenic torsion. Additionally, this patient also had a dolichocolon, a condition where the colon is elongated and more prone to abnormal positioning. The prevalence of dolichocolon ranges from 1.9% to 28.5% [21]. Our theory suggests that due to this dolichocolon, the excess colon at the level of the descending and sigmoid colon tilted forward. As a result, the entangled spleen also shifted forward, ultimately resulting in splenic torsion and a dark, necrotic appearance. This splenic torsion was the reason for the patient’s diffuse abdominal pain. As mentioned above, the colon was also tilted forward, which resulted in volvulus (twisting of the colon) and eventually led to partial obstruction. This partial obstruction caused aerocoly, contributing to the distended abdomen observed during physical examination [22].

There are three key insights to be obtained from this case report. Firstly, it emphasizes the complexity of symptomatology, where multiple factors can contribute to a single symptom, such as abdominal pain. Therefore, clinical reasoning remains essential. While sporadic torsion and detorsion of the ovarian fibroma likely caused the pain in both iliac regions, they do not explain the diffuse abdominal pain or abdominal distention in this case. Thus, a critical approach is necessary to identify the cause of each specific symptom, as one pathology may not always fully account for all observed symptoms. Secondly, this case report underscores the importance of multidisciplinary collaboration within a surgical setting. The consultation between the operating gynecologist and the abdominal surgeon in this case highlights the significance of such cooperation. Without this approach, the splenic torsion might have remained unresolved, resulting in splenic infarction and necessitating splenectomy. Thirdly, this report highlights the importance of adhering to a standardized procedure during laparoscopy, which involves assessing the morphology, location, size, and color of all abdominal organs. Consistent evaluation of abdominal organs via laparoscopy enables familiarity with their normal appearance, aiding in the identification of abnormalities, such as the abnormal spleen observed in this case report.

## Conclusions

This case report illustrates the rare co-occurrence of an ovarian fibroma alongside a concurrent splenic torsion, presenting as an acute abdomen. Ovarian fibromas and splenic torsions both often present with nonspecific symptoms, making diagnosis challenging. Early detection and intervention are essential for optimal outcomes. The complexity of symptomatology emphasizes the importance of thorough clinical assessments and additional investigations in diagnosing such cases.

This case highlights the importance of prompt diagnosis and interventions in the management of complex abdominal pathologies, ultimately aiming to improve patient outcomes and mitigate potential consequences.
